# Retraction

**DOI:** 10.1111/jcmm.16033

**Published:** 2021-01-04

MicroRNA‐140‐5p elevates cerebral protection of dexmedetomidine against hypoxic–ischaemic brain damage via the Wnt/β‐catenin signalling pathway. Xin‐Rui Han, Xin Wen, Yong‐Jian Wang, Shan Wang, Min Shen, Zi‐Feng Zhang, Shao‐Hua Fan, Qun Shan, Liang Wang, Meng‐Qiu Li, Bin Hu, Chun‐Hui Sun, Dong‐Mei Wu, Jun Lu, Yuan‐Lin Zheng. J Cell Mol Med. 22: 3167‐3182 (https://doi.org/10.1111/jcmm.13597).

The above article from Journal of Cellular and Molecular Medicine, published online on 13 March 2018 on Wiley Online Library (wileyonlinelibrary.com) in Volume 22, pp. 3167‐3182, has been retracted by agreement between the authors, the journal Editor‐in‐Chief and John Wiley & Sons Ltd. The retraction has been agreed following an investigation based on allegations raised by a third party. The authors asked to retract this manuscript as raw experimental data were not accessible anymore and thus the validity of the overall results could not be confirmed.

